# Assessment of Sub-micrometer-Sized Particles with Practical Activities in an Underground Coal Mine

**DOI:** 10.1007/s42461-024-01140-w

**Published:** 2024-11-21

**Authors:** Yi-Hsuan Chen, Alejandro Munoz, Connor Krause, Jürgen Brune, Candace S. J. Tsai

**Affiliations:** 1 Department of Environmental Health Sciences, University of California, Los Angeles, Los Angeles, CA USA; 2 Mining Engineering Department, Colorado School of Mines, Golden, CO USA

**Keywords:** Miner exposure, RCMD, Respirable particles, Sampling technique

## Abstract

This assessment was designed to explore and characterize the airborne particles, especially for the sub-micrometer sizes, in an underground coal mine. Airborne particles present in the breathing zone were evaluated by using both (1) direct reading real-time instruments (RTIs) to measure real-time particle number concentrations in the workplaces and (2) gravimetric samplers to collect airborne particles to obtain mass concentrations and conduct further characterizations. Airborne coal mine particles were collected via three samplers: inhalable particle sampler (37 mm cassette with polyvinyl chloride (PVC) filter), respirable dust cyclone (10 mm nylon cyclone with 37 mm Zefon cassette and PVC filter), and a Tsai diffusion sampler (TDS). The TDS, a newly designed sampler, is for collecting particles in the nanometer and respirable size range with a polycarbonate filter and grid. The morphology and compositions of collected particles on the filters were characterized using electron microscopy (EM). RTIs reading showed that the belt entry had a greatly nine-times higher total particle number concentration in average (~ 34,700 particles/cm^3^) than those measured at both the underground entry and office building (~ 4630 particles/cm^3^). The belt entry exhibited not only the highest total particle number concentration, but it also had different particle size fractions, particularly in the submicron and smaller sizes. A high level of submicron and nanoparticles was found in the belt conveyor drift area (with concentrations ranging from 0.54 to 1.55 mg/m^3^ among three samplers). The data support that small particles less than 300 nm are present in the underground coal mine associated with dust generated from practical mining activities. The chemical composition of the air particles has been detected in the presence of Ca, Cu, Si, Al, Fe, and Co which were all found to be harmful to miners when inhaled.

**Supplementary Information:**

The online version contains supplementary material available at https://doi.org/10.1007/s42461-024-01140-w.

## Introduction

Exposure to coal mine dust has been recognized as a hazard for decades. Unlike engineered particles [22, 24, 33] and the naturally emitted ambient particles [7, 9, 15], sub-micrometer- and nanometer-sized particles in the coal mining environment represent a potentially serious but largely unstudied exposure risk [3, 21]. Modern mining developed in recent decades uses larger and more powerful mining machines, thus efficiently increasing the productivity of coal extraction, along with an increased amount of dust generated. The dust can contain a high proportion of very small particles.

In the coal mining industry, sub-micrometer particles broken down from rocks when cutting, crushing, or explosions occurred in the mining environment have been reported since the 1950s (Brown et al., 1950). Respirable coal mine dust (RCMD), having an aerodynamic diameter < 10 μm and a median cut-point (d_50_) of 4 μm, is generated during mining operations as a byproduct [4]. Inhalation of mineral dust clouds containing RCMD and especially those containing quartz (crystalline silica) has been strongly associated with occupational pulmonary diseases, including coal worker’s pneumoconiosis (CWP), silicosis, mixed dust pneumoconiosis, dust-related diffuse fibrosis (DDF), and progressive massive fibrosis (PMF) [16, 25, 32].

Previous investigations have shown that exposures related to some underground tasks may exceed the permissible exposure limit (PEL) of respirable coal and silica dust. Despite years of effort to understand and reduce CWP prevalence from more than 30% in 1970 to less than 4.2% in the late 1990s, the prevalence among the USA coal miners has unexpectedly increased since then [10, 11, 19, 29]. During the mid-2010s, the CWP trend started to change, presenting more severe CWP cases [17, 25, 27]. Recent investigations have found a high concentration of fine aerosols (< 100 nm) occurs in underground coal mine places, especially during the operation of mining machines [27, 28]. Mine Safety and Health Administration (MSHA) reduced the PEL of coal dust from 2.0 to 1.5 mg/m^3^ on August 1, 2016, requiring sampling for the full duration of the miner’s shift. Identifying the cause of the drastic rise of CWP among the miners remains a challenge based on limited knowledge of RCMD exposure in the atmosphere. The challenge is not only discussed recently in a consensus study report of the National Academies of Sciences, Engineering, and Medicine but also noted it in a NIOSH document [5, 17]. Additionally, the need for future research to clarify the relationship between RCMD characterization and occupational lung disease is emphasized by researchers [20, 23, 25, 32].

For the purpose of evaluating miners’ exposure to coal mine dust, MSHA requires mine operators to routinely measure the mass concentration of breathing zone respirable dust. This evaluation is usually conducted by measuring exposure concentrations with gravimetric filter sampling or direct reading devices, i.e., personal dust monitor (PDM) for evaluation in the coal mine, to obtain total mass concentrations. The available method for exposure evaluation cannot sufficiently quantify the mass of specific particle sizes or those smaller than one micrometer owing to their trace amount of mass. Due to the design and limitation of the MSHA-approved device (PDM), it has been challenging to characterize very fine mining dust particles. Additionally, the precision of using mass-based monitoring and sampling methods is questionable in identifying the contribution of these small particles to the overall RCMD [17].

Thus, this field study is aimed at evaluating and characterizing respirable particles specifically for those ranging from sub-micrometer- to nanometer-sized particles emitted from mining activities in an underground coal mine. A newly designed sampler, the Tsai Diffusion Sampler (TDS) [31] for sampling and characterizing nanoparticles and respirable particles, was used side by side with other sampling and measurement techniques for this evaluation.

## Methods

**Fig. 1:**
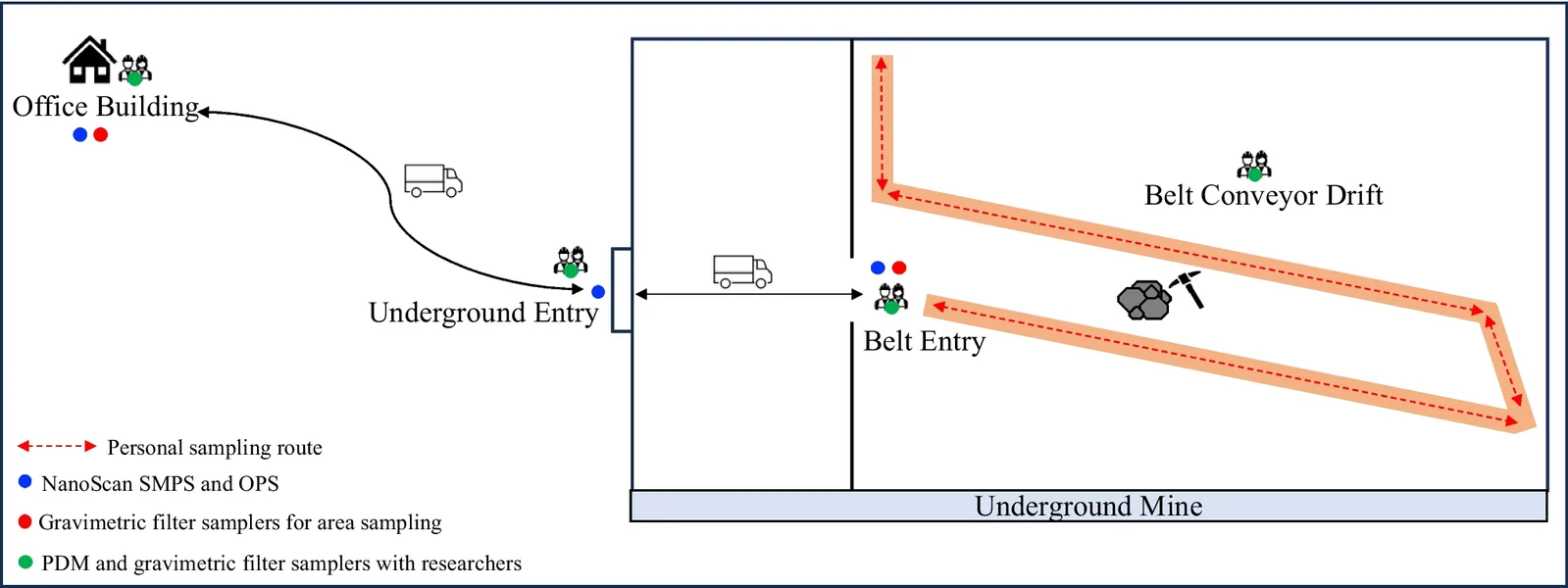
The underground mine with task locations and sampling design

This study was conducted at an underground coal mine in the USA. The mine dust particle exposure was continuously assessed and monitored using direct reading real-time instruments (RTIs). Multiple samplers were applied to collect airborne coal mine dusts including an inhalable particle sampler, a respirable dust cyclone, and the TDS. The underground mine areas to be evaluated were the office building, an underground entry, a belt entry, and a belt conveyor drift area, which represent the work environments with different work tasks. Figure 1 illustrates the relative sampling locations with the employed methods and associated tasks at the mine site.

### Direct Reading Real-Time Measurements

RTIs used for this study include a NanoScan scanning mobility particle spectrometer (SMPS) (model 3910, TSI Incorporated, Shoreview, MN, USA), an optical particle sizer (OPS) (model 3330, TSI Incorporated, Shoreview, MN, USA), and a PDM (model 3700, Thermo Scientific™, Waltham, MA, USA). Real-time total dust number concentrations in particle counts were retrieved from NanoScan, SMPS, and OPS and averaged to present the particle levels in the underground mine areas. All averaged number concentrations are expressed in particles/cm^3^, along with the standard deviation ( ±) showing the range. The NanoScan SMPS has a measurement range of 10–420 nm particle level, with a recommended maximum concentration measurement of 1 × 10^6^ particles/cm^3^. The OPS has a range of 0.3–10 μm, with a recommended maximum concentration measurement of 3000 particles/cm^3^. Proven underground capable, the PDM-3700 personal dust monitor offers continuous personal respirable dust exposure information. The office building, underground entry, and belt entry work environments were measured using NanoScan, SMPS, and OPS to assess particle concentrations. The PDM was worn by the researchers to measure the personal, respirable-sized, particle mass concentrations during the entire monitoring period starting from riding the vehicle from the office building to the belt entry, during the entire period walking along the belt conveyor drift, and the ride returning to the office building.

### Gravimetric Filter Sampling

**Fig. 2:**
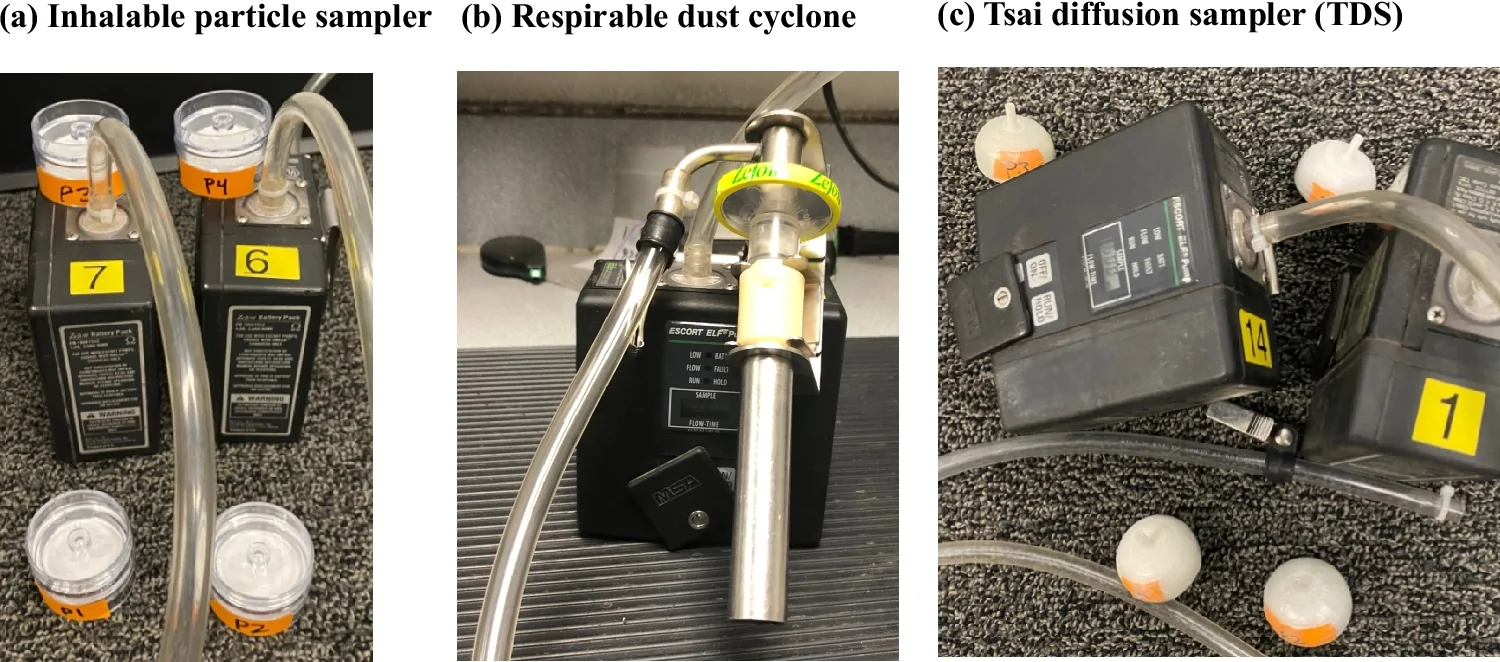
Samplers used in the coal mine field. **a** Inhalable particle sampler (37 mm cassette with PVC filter) (NIOSH0500), **b** respirable dust cyclone (10 mm nylon cyclone with 37 mm Zefon cassette and PVC filter), and **c** TDS (25 mm PVC filter)

**Fig. 3:**
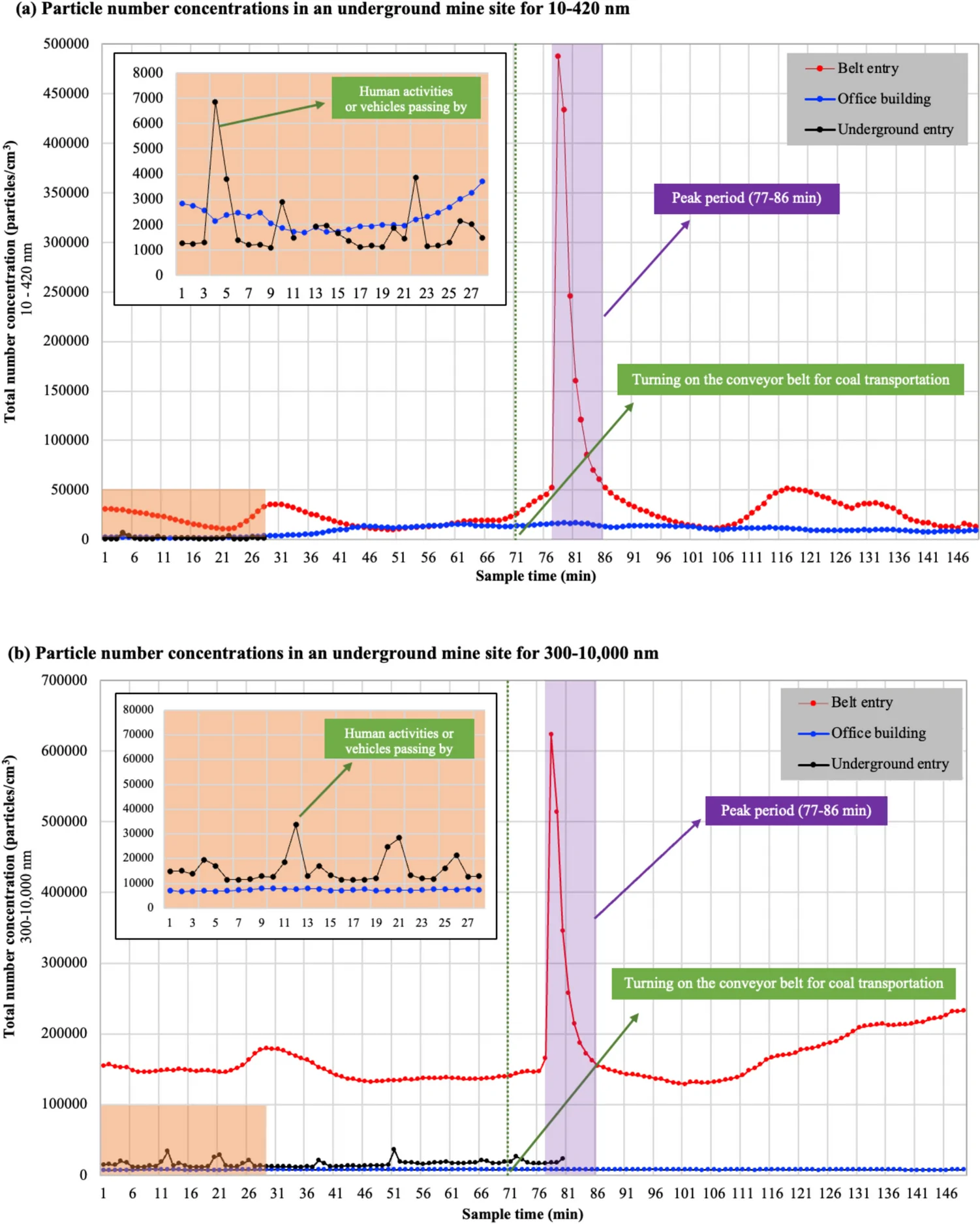
Area particle sampling in an underground mine site on the office building, underground entry, and belt entry by **a** NanoScan SMPS and **b** OPS

Airborne mine particles were collected using three different samplers: (1) inhalable particle sampler: a 37 mm cassette with a polyvinyl chloride (PVC) filter according to the NIOSH0500 sampling method (Fig. 2a), (2) respirable dust cyclone: a 10 mm nylon cyclone with 37 mm Zefon cassette and PVC filter approved by the MSHA (Fig. 2b), and (3) a 25 mm TDS (Fig. 2c) which collected particles on the polycarbonate filter and transmission electron microscope (TEM) grid. The particle collection includes three representative locations of area samples (office building, belt entry, and belt conveyor drift area) and two personal samples each attached onto research team members for breathing zone sampling. Four research scientists were walking in two groups, the first group 100–150 m ahead of the second, to evaluate the difference in particle exposure through disturbing the settled mine dust on the ground. Triplicated samples of each sampler were taken, except for the PDM measurement.

The filter samples from the inhalable particle sampler, respirable dust cyclone, and TDS were analyzed via gravimetric weighing using a balance (Mettler-Toledo MX5, Columbus, OH, USA) to determine the mass collected and to estimate the mass concentration. Blank filters and field sampling filters were separately placed in the weighting chamber located in the balance room overnight before weighing. Each of the pre-sampling and sampled filters were weighed three times and results were averaged. All averaged mass concentrations are expressed in mg/m^3^, along with the standard deviation ( ±) showing the range.

### TDS Sampler

The TDS is designed for collecting particles in the nanometer and respirable size range [13, 31]. This novel design has been published and applied in several occupational sampling-based research [12, 13, 18]. It operates at a flow rate of 0.3 L/min and directly collects particles onto a polycarbonate membrane filter (25 mm diameter, 0.22 μm pores). A TEM grid is attached at the center of the filter. The low airflow (0.3 L/min) sampling operated in TDS enhances the collection of nanoparticle/ultrafine particles as a result of strong Brownian motion and laminar airflow entering the cassette and flowing through the polycarbonate filter. Respirable particles and nanoparticles collected on the filter and the grid were further characterized. The collection efficiency of the TDS has been established experimentally and follows a sigmoidal curve with a cut point diameter (d_50_) of approximately 3.8 μm, expressed as the mass median aerodynamic diameter [31]. This cut point closely mirrors the respirable fraction cut point diameter (d_50_ = 4 μm) given by the particle size-selective sampling criteria for airborne particulate matter established by the American Conference of Governmental Industrial Hygienists (ACGIH) [1]. The inlet diameter, sampler geometry, and operating flow rate result in the collection of a size-selective sample. The TDS allowed the mass concentration of respirable particles to be obtained while also enabling the characterization of nanoparticles using TEM or scanning electron microscopy (SEM).

### Image Analysis

The particles collected on the grids in TDS were analyzed via TEM at 200 kV (JOEL JEM-2100F, JOEL, Peabody, MA, USA). The particles collected on the polycarbonate filter in TDS and PVC filter in 37 mm cassette were examined by SEM at 15 kV (JOEL JSL-6500F, JOEL, Peabody, MA, USA) to evaluate the morphology representing the relative fine and coarse size portion of particles. Additionally, the elemental composition of the TEM grid samples was analyzed by energy dispersive spectroscopy (EDS) on the TEM.

## Results and Discussion

Tables 1 and 2 summarize all concentration results including RTI measurements and gravimetric filter sampling of the underground mine dust particles. Data were discussed in the following sections.

**Table 1:** Summary of RTI detection for the measurement and analysis of underground mine dust particles

Instrument	Locations	Sampling time (min)	Sample analysis (outcomes)
NanoScan SMPS and OPS	Office building	926	Number concentration, size-fraction number concentration
	Belt entry	149	
	Underground entry	29	
PDM*	Office building	95	Mass concentration
	Belt entry	170	
	Underground entry	18	

^*^The entire time of walking was 89 min in the bel conveyor drift area. PDM was carried on by scientists and monitored continuously during the period

**Table 2:** Summary of filter sampling for the measurement and analysis of underground mine dust particles

Sampler	Locations	Sampling time (mins)	Dust weight (mg)	Volume (m^3^)	Concentration (mg/m^3^)
Inhalable particle sampler	Office building	525	0.02	0.525	0.02 ± 0.02
	Belt entry	210	0.03	0.210	0.12 ± 0.01
	Belt conveyor drift	147	0.77	0.148	5.18
	Personal	62	0.07	0.062	1.10 ± 0.18
TDS	Office building	407	0.05	0.407	0.16 ± 0.08
	Belt entry	210	0.02	0.210	0.09 ± 0.10
	Belt conveyor drift	147	0.18	0.148	1.23 ± 1.04
	Personal	62	0.03	0.062	0.54 ± 0.03
Respirable dust cyclone	Office building	527	0.12	0.53	0.42 ± 0.33
	Belt entry	210	0.14	0.21	0.67 ± 0.41
	Belt conveyor drift	147	0.20	0.15	1.35 ± 0.30
	Personal	62	0.09	0.06	1.55 ± 2.18

### Mine Dust Particle Exposure Level Measured with Direct Real-Time Reading Instruments

Figure 3 displays the total particle exposure represented by number concentration throughout the entire monitoring period measured by NanoScan SMPS and OPS in the office building, underground entry, and belt entry at the mine site. For particles in the submicron ranges measured by NanoScan SMPS, the belt entry (34,700 ± 56,300 particles/cm^3^) had a much higher total particle number concentration than the underground entry (4180 ± 12,300 particles/cm^3^) and office building (4630 ± 3830 particles/cm^3^). OPS data also supported a similar finding that concentrations in the belt entry (167,000 ± 57,700 particles/cm^3^) are higher with mine dust particles than those measurements in the office building (10,180 ± 1960 particles/cm^3^) and underground entry (16,100 ± 4760 particles/cm^3^), as can be seen in Fig. 3b. Compared to the relatively stable office environment, the fluctuation of concentrations at the underground entry (at around 6–7, 14, and 24 min when sampling started) revealed that particle exposure may have been related to human activities, such as miners or vehicles passing by the entrance (Fig. 3a). The particle concentrations with peak and non-peak concentrations are presented separately in the embedded figure in Fig. 3a and b. These activities, disturbing dust on the ground and on the surface of vehicles, contributed to the high peaks of particle number concentration at underground entry, which elevated the exposure level from 1600 ± 632 particles/cm^3^ to 20,270 ± 30,880 particles/cm^3^ detected by NanoScan SMPS. A similar increase is observed in OPS data, presented that the particle number concentration detected in the underground entry was elevated from 14,800 ± 2620 particles/cm^3^ to a higher level of 25,800 ± 5230 particles/cm^3^, apparently at similar time periods as measured by NanoScan SMPS. At the belt area, NanoScan SMPS and OPS both showed peak concentrations occurred around 80 min and quickly returned to the stable level after 15 min (Fig. 3). The increase was contributed from a large amount of dust resuspended by the vibration with regard to turning on the conveyor belt for coal transporting.

When we researched the high peak period of number concentration at belt entry and compared it to the non-peak period, as well as other areas, the peak period at belt entry showed concentrations ranging from 52,300 to 488,000 particles/cm^3^ for NanoScan SMPS and 155,000–624,000 particles/cm^3^ for OPS. The non-peak period at the belt entry was 10,460–51,600 particles/cm^3^ for NanoScan SMPS and 129,000–232,000 particles/cm^3^ for OPS. During the non-peak period in the belt entry measurements, the averaged total particle number levels remained high on both NanoScan SMPS (24,400 ± 11,300 particles/cm^3^) and OPS (159,000 ± 28,400 particles/cm^3^), which were much higher than the other areas (office building and underground entry) regardless of whether or not the site was transporting the coal on the belt. The differences in peak to non-peak concentrations demonstrate that a very high level of submicron-sized particles would easily be generated from the belt transporting coal. The submicron particles would be suspended in the air for a longer period of time compared to their larger micrometer-sized particles [8, 14]. The mining activities such as coal transporting on the conveyor belt and miners walking or performing jobs would continuously generate high levels of sub-micrometer-sized particles and potentially lead to a high risk of developing lung disease among the miners under long-term exposure.

**Fig. 4:**
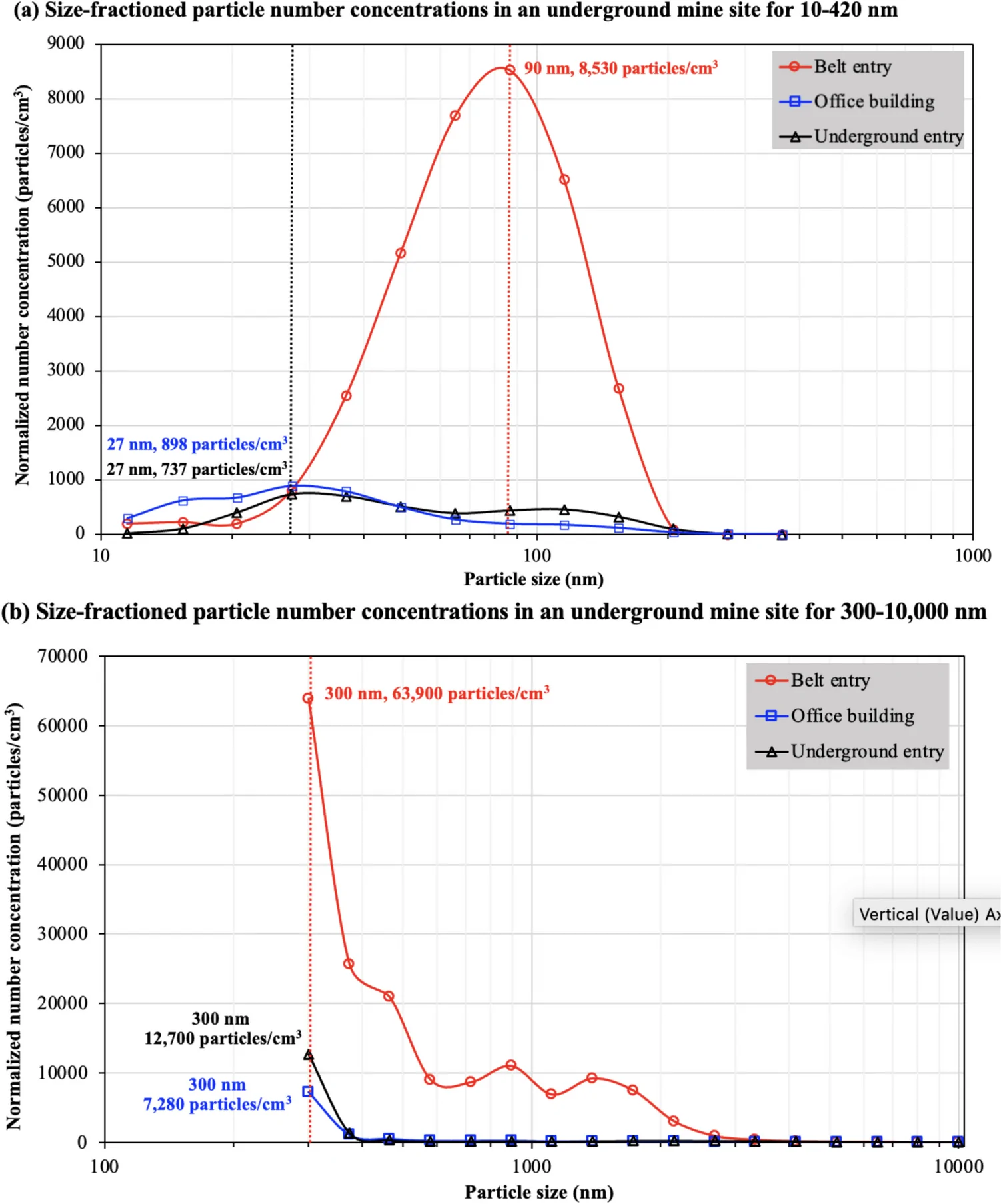
Size-fractioned particle number concentrations in an underground mine site on the office building, underground entry, and belt entry by **a** NanoScan SMPS and **b** OPS

Figure 4 presents the size fraction results measured by NanoScan SMPS and OPS. The results clearly showed that the belt entry exhibited the highest concentration across micron- to submicron-sized (0.3–3.3 μm) and nanosized (36.5–205.4 nm) particles compared to the office building and underground entry. However, more coarse particles (larger than 300 nm) were presented in the underground mine and its office. The OPS measured concentrations (Fig. 3) were 2.2 (office building), 4.8 (belt entry), and 3.9 (underground entry) times higher than NanoScan SMPS concentrations. Particularly for the measurements in the belt entry, the average concentration measured in 300–10,000 nm by OPS including micron- to submicron-sized particles was 9830 particles/cm^3^, and the submicron-sized particles (10–420 nm) measured by NanoScan SMPS were found to be in a much lower concentration of 2670 particles/cm^3^. The differences of concentrations between two RTIs measuring large (OPS) and small (NanoScan SMPS) size ranges in the office building and at the underground entry were not as obvious as in the belt entry. The belt entry’s highest concentration of 8530 particles/cm^3^ occurred at a particle size of 90 nm while both the office building (898 particles/cm^3^) and underground entry peaked at 27 nm (737 particles/cm^3^).

**Fig. 5:**
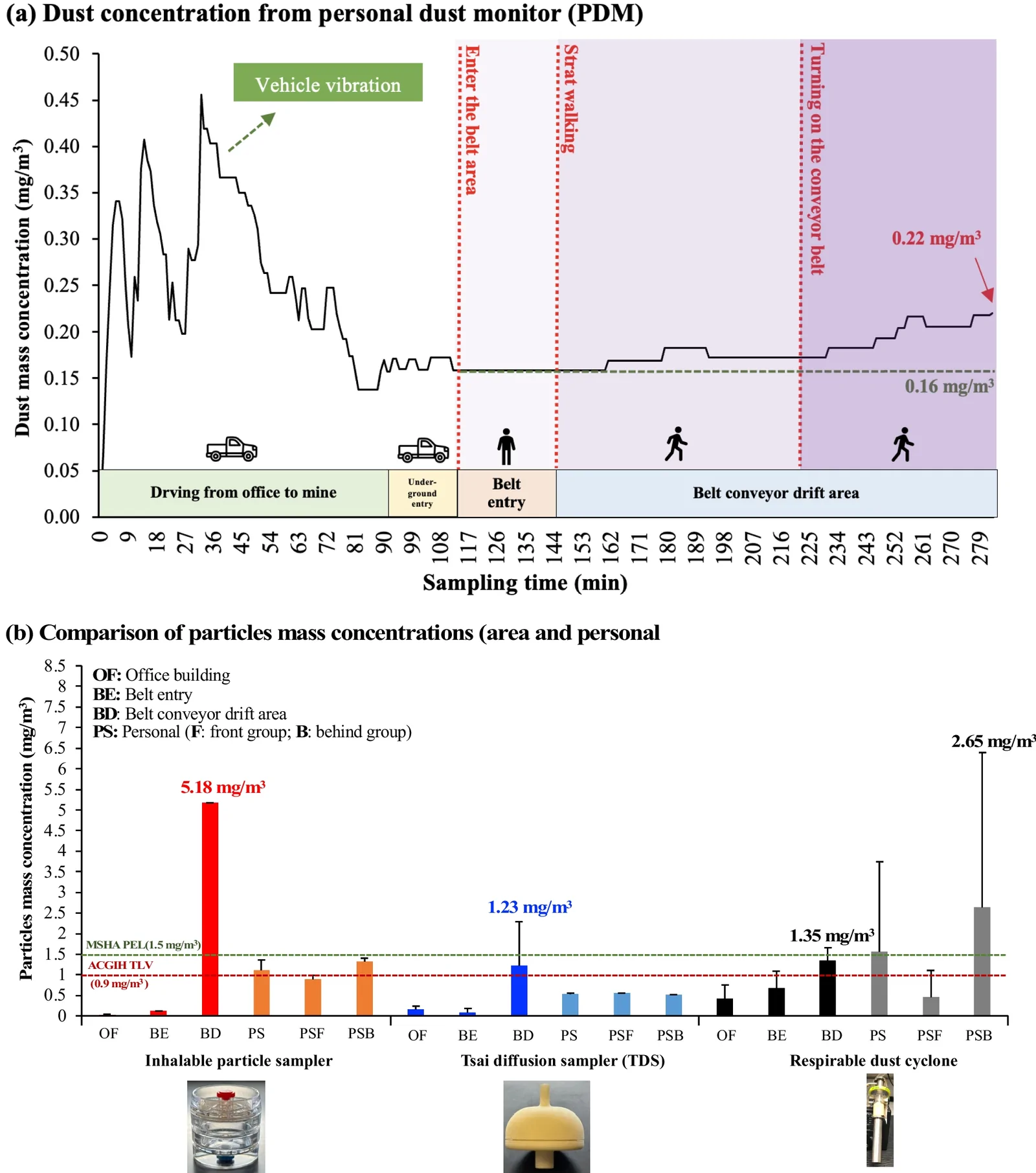
Mass concentrations presented the dust particle exposure in the mining industry at multiple locations (office building, belt entry, and belt conveyor drift area). **a** The PDM mass concentration monitored during the entire activity period. **b** Concentrations of three samplers collected at multiple locations. Results showed that the belt conveyor drift area has more respirable dust particles

PDMs were used to monitor the personal dust exposure in mass concentration levels in the mine together with the gravimetric samplers. Figure 5a shows the mass concentration monitored by PDM throughout the entire period of measurement, which could represent a daily routine of miners performing similar activities. The high level of fluctuation of particle concentrations during the period traveling underground into the mine (0.26 mg/m^3^) may have been caused by the vibration in the vehicle. The level dropped and remained at a more stable and lower level when reaching the belt conveyor drift area (0.16 mg/m^3^) and during the walk along the belt (0.18 mg/m^3^). The average mass concentration measured by PDM in the belt conveyor drift area was 0.18 ± 0.02 mg/m^3^. When the conveyor belt was running for coal haulage, the average mass concentration started to climb up (0.20 mg/m^3^) and reached the level of 0.22 mg/m^3^ at the end of PDM sampling.

### Mine Dust Particle Exposure Levels Evaluated with Samplers and Mass Concentrations

The measurement, sampling, and analysis by three samplers to collect airborne mine particles are summarized in Tables 1 and 2. The measured mass concentrations clearly show that more mine dust was collected in the belt conveyor drift area than in the office building and belt entry (Tables 1 and 2). The inhalable particle sampler and respirable dust cyclone were designed to sample respirable dust particles, and TDS was used for collecting particles in both the nanometer and respirable size range. The inhalable particle sampler using a cassette found that the belt conveyor drift area has the highest respirable particle level (5.18 mg/m^3^), followed by the belt entry (0.12 mg/m^3^), then the office building (0.02 mg/m^3^). The respirable dust cyclone also found the same difference in that the belt conveyor drift area has the highest respirable particle level at 1.35 mg/m^3^, followed by 0.67 mg/m^3^ at the belt entry and 0.42 mg/m^3^ at the office building. Collecting particles in the nanometer and respirable size range, the TDS sampler results supported the finding that the highest respirable particle exposure was in the belt conveyor drift area (1.23 mg/m^3^), compared to 0.09 mg/m^3^ at the belt entry and 0.16 mg/m^3^ at the office building.

Regarding personal exposure levels of two groups of team members walking at front and back, we found that personal sampling mass concentration by the NIOSH0500 (inhalable particle sampler) and cyclone methods show that the scientists walking in the second group were exposed to a much higher concentration of mine dust particles than those in the group walking in front of them, likely due to dust kicked off from the ground by the front group. However, the TDS sampled concentrations showed similar exposure levels of the team walking behind and in front, 0.52 mg/m^3^ and 0.56 mg/m^3^ respectively. This similarity of TDS sampler results is likely because TDS efficiently collected submicrons and nanoparticles in the respirable sized range and limited the heavy particles at the upper sizes of respirable particles. The TDS results also indicate that the dust kicked off from the ground by the front group might contain more larger particles which were collected more by inhalable particle sampler and respirable dust cyclone.

Mass concentrations from samplers were calculated to compare with ACGIH threshold limit value (TLV) and MSHA PEL as displayed in Fig. 5b. Referring to the ACGIH TLV-time-weighted average (TWA) (0.90 mg/m^3^) for respirable bituminous coal mine dust and MSHA PEL (1.50 mg/m^3^) at underground coal mines, although our mass concentration results were not an 8-h TWA, the dust exposure levels found in belt area are still more of a concern and the potential exposure to workers walking behind others required more attention on the possible elevated exposure levels. The particle mass concentrations at the belt conveyor drift area were found to be different with different sampling methods, i.e., 5.18 mg/m^3^ by cassette, 1.23 ± 1.05 mg/m^3^ by TDS, and 1.35 ± 3.74 mg/m^3^ by cyclone. As a result, three types of personal samplers sampled at the belt conveyor drift area all have a much higher exposure to mine dust particles than the office building (0.02, 0.16, and 0.42 mg/m^3^) and belt entry (0.12, 0.09, and 0.67 mg/m^3^).

### Morphology and Composition Characteristics of Particles

**Fig. 6:**
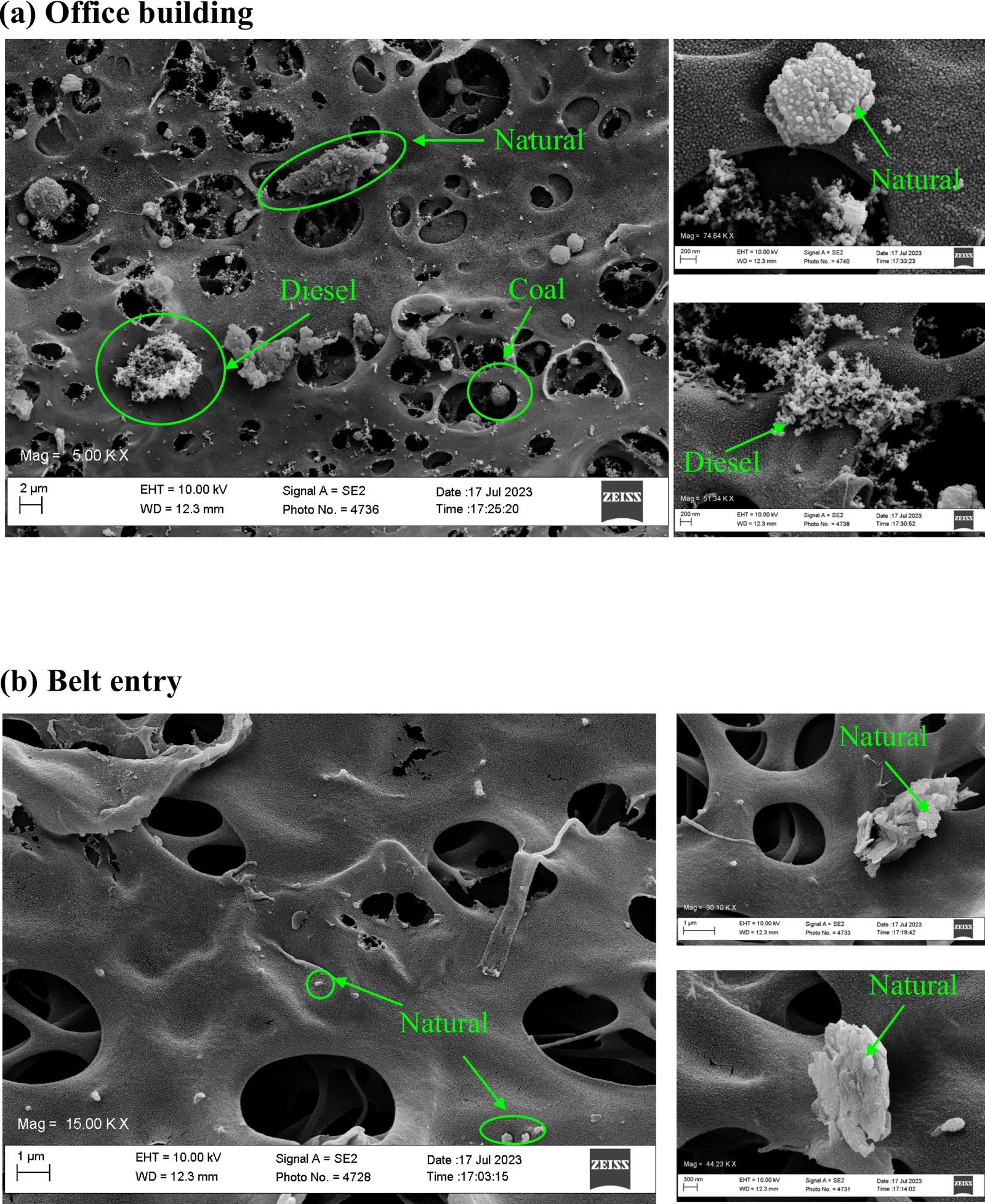

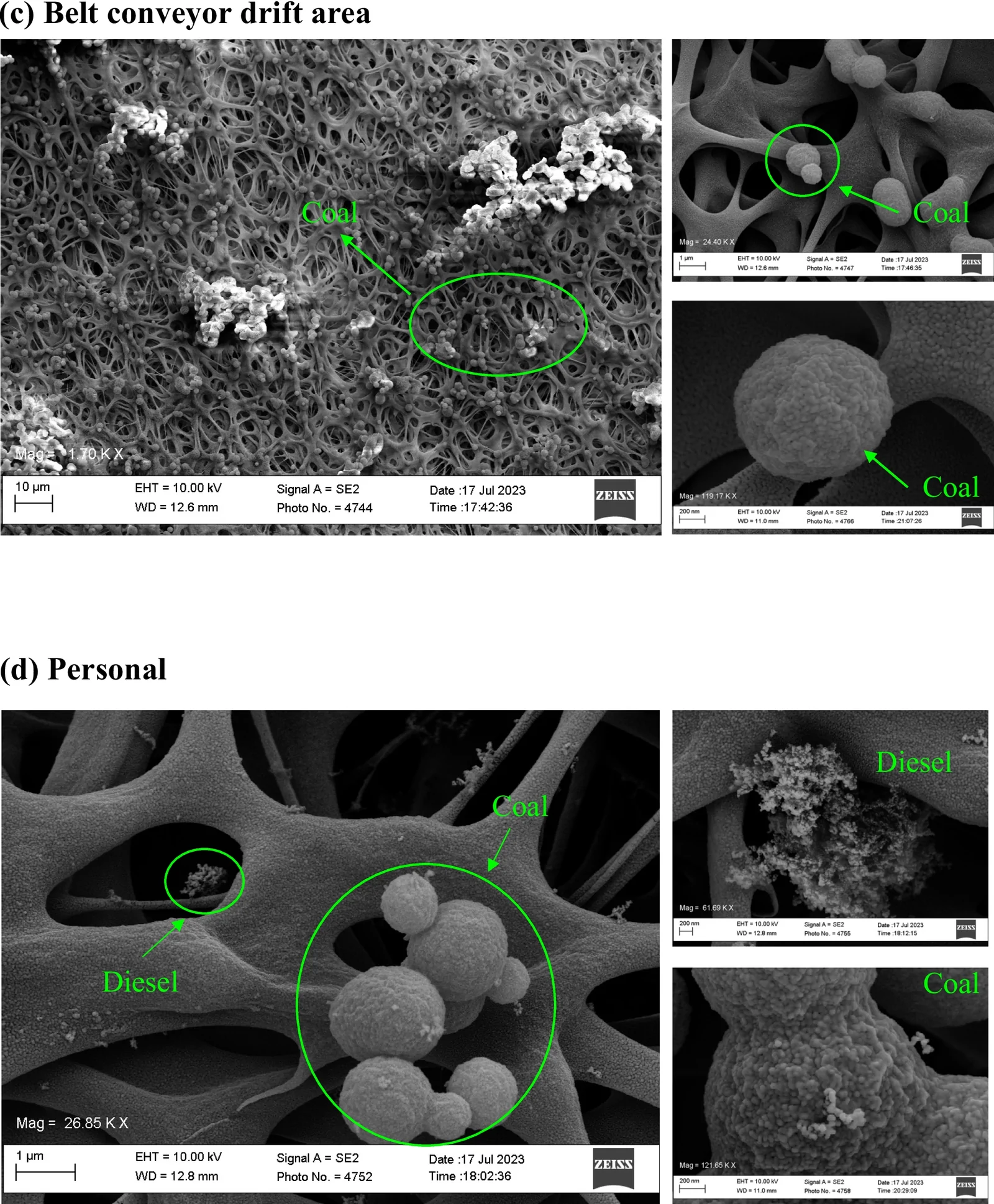
Electron microscope images of dust particles sampled by inhalable particle sampler (37 mm cassette with PVC filter) (NIOSH0500 method) at the underground mine present the particulate morphology in multiple locations by SEM. **a** Office building. **b** Belt entry. **c** Belt conveyor drift area. **d** Personal at belt conveyor drift area

Surface features and morphology of dust particles collected by the samplers were analyzed by electron microscope, i.e., SEM and TEM. The SEM images present the morphology of particles which differ at various sampling locations. In the office building, the particles were relatively big (> 2 μm), and coal and diesel exhaust particles (marked with arrows showing coal and diesel exhaust particles) were observed indicating the office building was contaminated with mine dust particles which could be carried by personnel accessing the mine (Fig. 6a).

At the entrance to the belt conveyor drift, we found that the sampled particles by TDS sizes were relatively small compared to the particles collected in the office building and the morphology was monotonous (Fig. 6b). The SEM images remarkably presented extremely dense particle deposition on the TDS filter collected at the belt conveyor drift (Fig. 6c) and the personal samples taken while walking along the belt conveyor drift (Fig. 6d). At the belt conveyor drift area, mine dust particles were agglomerated together as spherical shape acclimating on the filters. The sizes range from approximately 400 nm to 1 μm (Fig. 6c). Differing from the solid structure of particles at the belt conveyor drift area, the surface of agglomerated particles was loose, and more fiber-shape particulates were noticed in the personal samples, with size finer than 200 nm (Fig. 6d). Diesel exhaust particles were also observed in the personal samples, referring to the images in a previously published study (Fig. 5d of referred study [30]) from diesel exhaust particles emitted from diesel locomotive operation [30]. Based on the S/TEM with EDS spectrum, detected metal elements (Pt, Co, Mg, Na, and Ni) (SI Figure S1) on these diesel-like particles showed much higher intensity than those similar elements in the coal or rock particles. This result supported that the particulates observed in Fig. 6d were from diesel exhaust emissions.

**Fig. 7:**
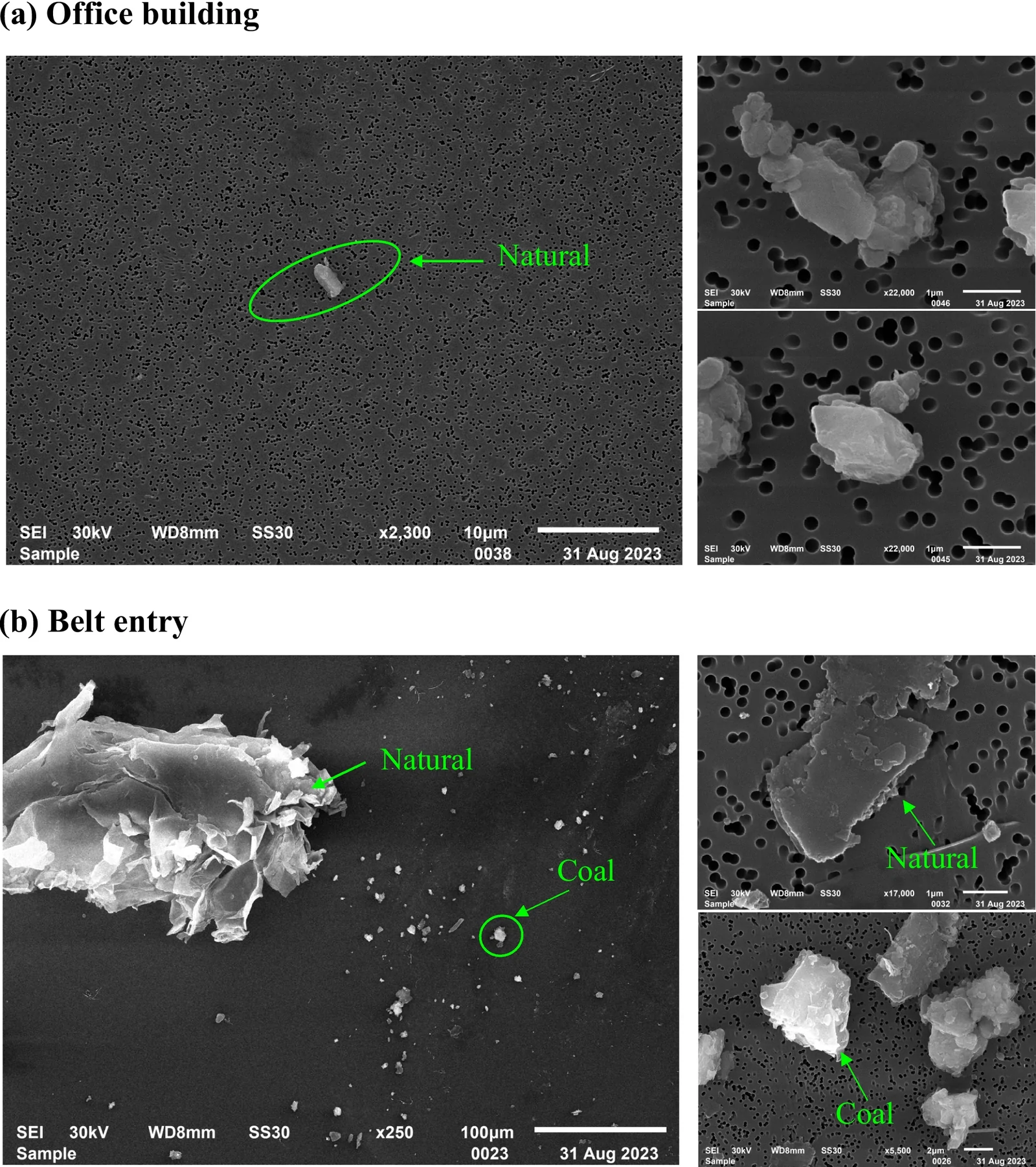

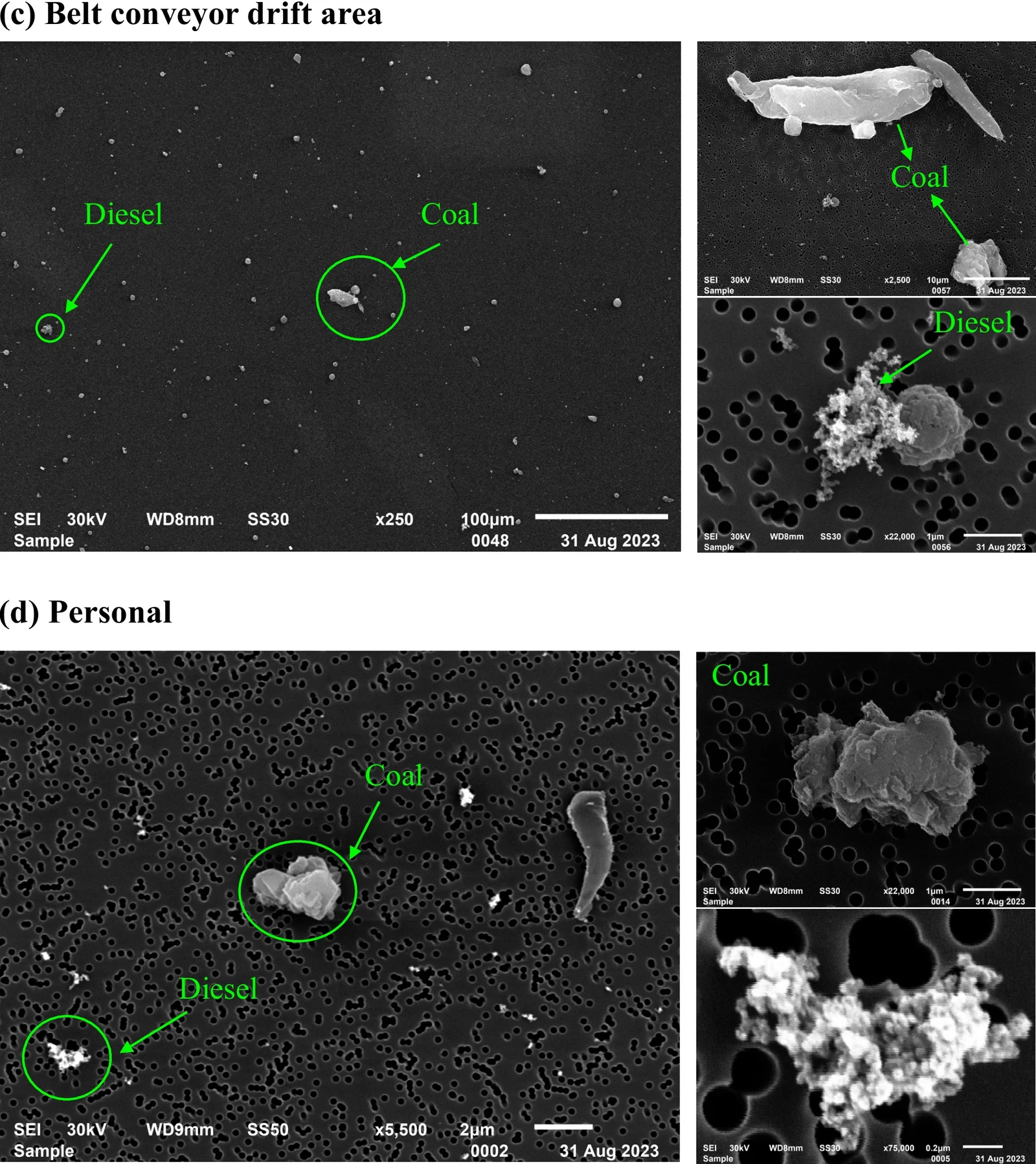
Electron microscope images of dust particles sampled by TDS with polycarbonate filters at the underground mine showing the particulate morphology at multiple locations. **a** Office building. **b** Belt entry. **c** Belt conveyor drift area. **d** Personal at belt conveyor drift area

Figure 7 presents the TEM images of dust particles sampled by TDS with polycarbonate filters at the underground mine with multiple locations and supports the finding about particulate morphology and emission sources. The particles collected in the office building as seen in Fig. 7a were found to be above 2 μm which were considered large and likely were carried in through humans and caused contamination in the office room. Mine dust and natural dust particles were both noticed on the samples collected at the belt entry as marked with arrows in Fig. 7b. Additionally, we found consistent results on the collected particles and the RTIs measurement. Area and personal samples collected at the belt area (belt entry and belt conveyor drift area) were observed with a high amount of mine dust particles, including very small nanoparticles including those smaller than 50 nm as seen in Fig. 7c and d.

**Fig. 8:**
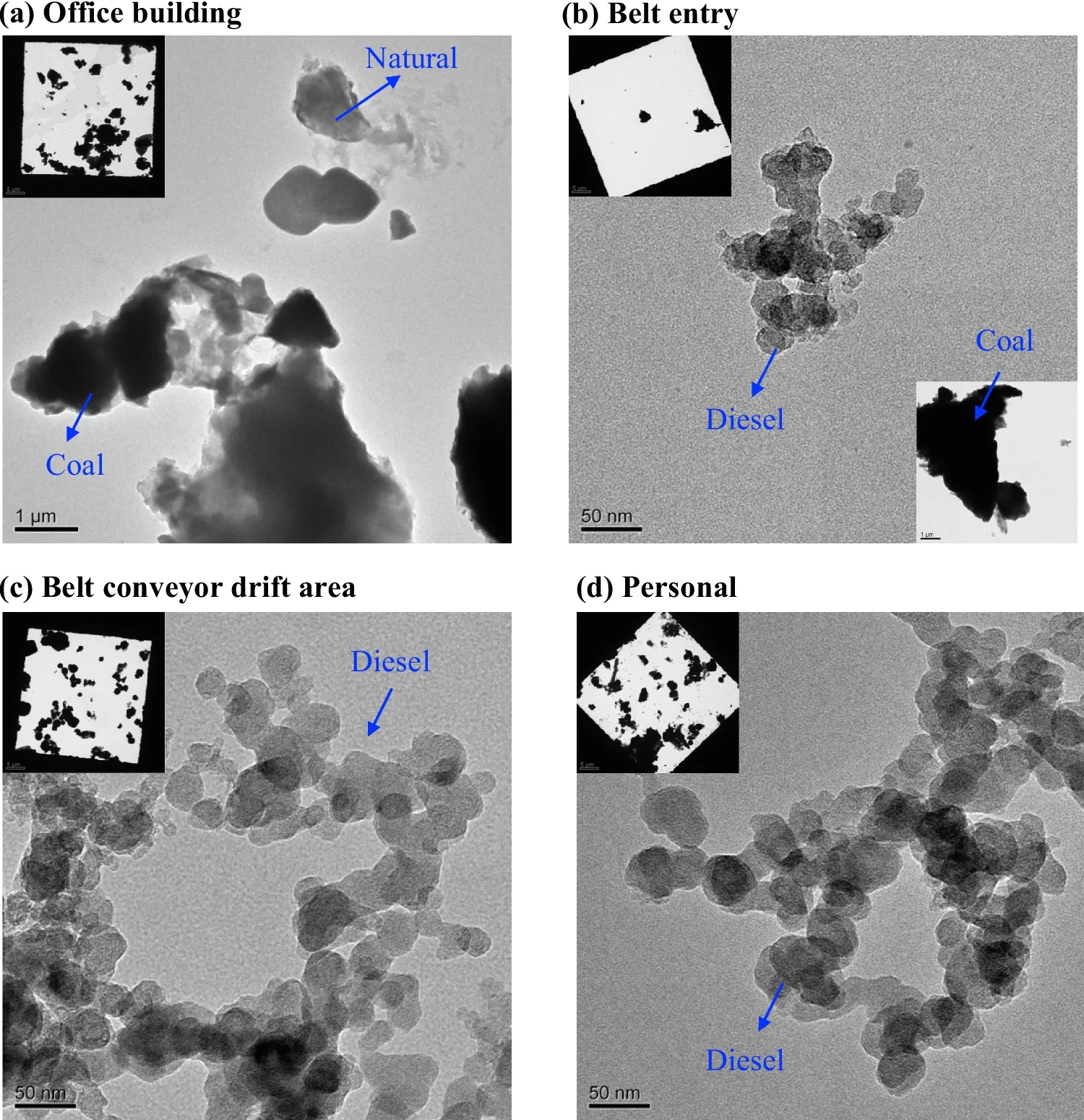
Transmission electron microscopy (TEM) images of dust particles deposited onto TEM grids sampled by TDS at the underground mine showing the particulate morphology at multiple locations. **a** Office building. **b** Belt entry. **c** Belt conveyor drift area. **d** Personal at belt conveyor drift area

The elemental composition of collected samples was examined by S/TEM with EDS spectrum. The results are shown on Figure SI1 with the spectrum map and element percentages. The elemental composition of particles in the office building confirmed the sources of particles which likely were from natural sources based on the presence of calcium, likely from the calcium carbonate rock dust used for inertization of coal dust. The absence of carbon, a major element in coal mines, supported the TEM finding (Figs. 6a and  7a), showing that the particle sources in the office environment were majority from the ambient environment and general dust carried by employees. The inorganic components are the majority of elements found in the office samples, including Ca (52.6%) followed by Cu (35.4%), Si (4.4%), Al (3.3%), Fe (3.2%), and Co (1.2%), which are similar to soil or earth crust [2]. The compositions of particles at the belt entry were similar to the office with Cu (87.0%) followed by Si (7.7%), Al (2.5%), Ca (1.2%), and Fe but were also detected other elements, like Cl and K. The elements found in the area and personal samples at the belt conveyor drift area were comparable, mostly consisting of C and Cu. According to our prior publication and results presenting the elemental compositions of coal and rock particles crushed from bulk coal and bulk rock [6], we can draw the conclusion that similar coal and rock particles were found on the samples collected in the belt conveyor drift area and personal breathing zone samples. Particles found in the personal samples carry more metal elements, including Pt (1.6%), Co (1.1%), Mg (0.5%), Na (0.4%), and Ni (0.2%), with most being toxic leading to a potential risk of adverse respiratory effect after inhalation (Fig. 8).

## Conclusions

The underground mine particles evaluated in this study showed the potential risk of exposure to submicrons and nanoparticles among miners. The RTI results clearly showed that the belt entry exhibited the highest concentration across all size fractions compared to the office building and underground entry, and a high level of submicrons and nanoparticles was found. Results from three filter samplers conclude that the respirable coal and mine particles (with concentrations ranging from 0.54 to 1.55 mg/m^3^) contributed to overexposure sometimes, and the workers walking behind others would suffer from a higher level of dust exposure than the person walking in front. These airborne particles found at the belt conveyor drift area contained various metal elements which might be toxic to human body. Miners who work in a similar mining environment and perform their daily activity in the underground coal mine could experience similar exposure as reported in this study. Other studies have shown that miners’ exposure is associated with RCMD from coal mine seam and surrounded rock strata, intake air, diesel exhaust, mining operations, and rock dusting [2, 26, 34].

In addition, the discrepancy between PDM measurement and gravimetric filter samplers seen in our sampling results is of concern. PDM was primarily designed to give real-time, continuous mass concentration for monitoring personal particle exposure in the mine environment. Unfortunately, the mass-based design limits its ability to represent the submicron and nanoparticle levels and results in an underestimated concentration of submicron-sized particles in the mining environment. The TDS, inhalable particle sampler (37 mm cassette), and respirable dust cyclone personal sampling results showed the breathing zone concentrations ranging from 1.23 to 5.18 mg/m^3^ when the persons walked in the belt conveyor drift area; however, the PDM presented a much lower level (0.18 ± 0.02 mg/m^3^) than our three gravimetric filter samplers.

To summarize, this field study concludes that RCMD generated in the underground mine with current mining extraction technology is still of concern with currently employed controls and practices. Based on our results and other studies, the sub-micrometer-sized particles in the mining are underestimated by the currently employed mass-based measurements. The protection for miners against exposure to small respirable particles must be prioritized to reduce the increased prevalence of CWP. In conclusion, our field study provided pioneering information using advanced and novel sampling devices for quantifying submicron portion of respirable particles, suggesting the possibility to apply the method for future particle exposure evaluation. The finding also will support other studies in a similar coal mining environment involving biological effects or disease development caused by submicrons and nanoparticles.

## Supplementary Information

Below is the link to the electronic supplementary material.

Supplementary file1 (PDF 697 KB)

## Data Availability

The data used to support the findings of this study are included within the article.
