# Supplementary material for: Assessment of Sub-micrometer-Sized Particles with Practical Activities in an Underground Coal Mine
Source: Min Metall Explor. 2024 Nov 21;41(6):2801–15. doi: 10.1007/s42461-024-01140-w (PMC11663185; doi:10.1007/s42461-024-01140-w)
Supplement: Supplementary file 1 — Supplementary file1 (PDF 697 KB) [file 42461_2024_1140_MOESM1_ESM.pdf]

## Supplementary Information (SI)

### (a) Office building

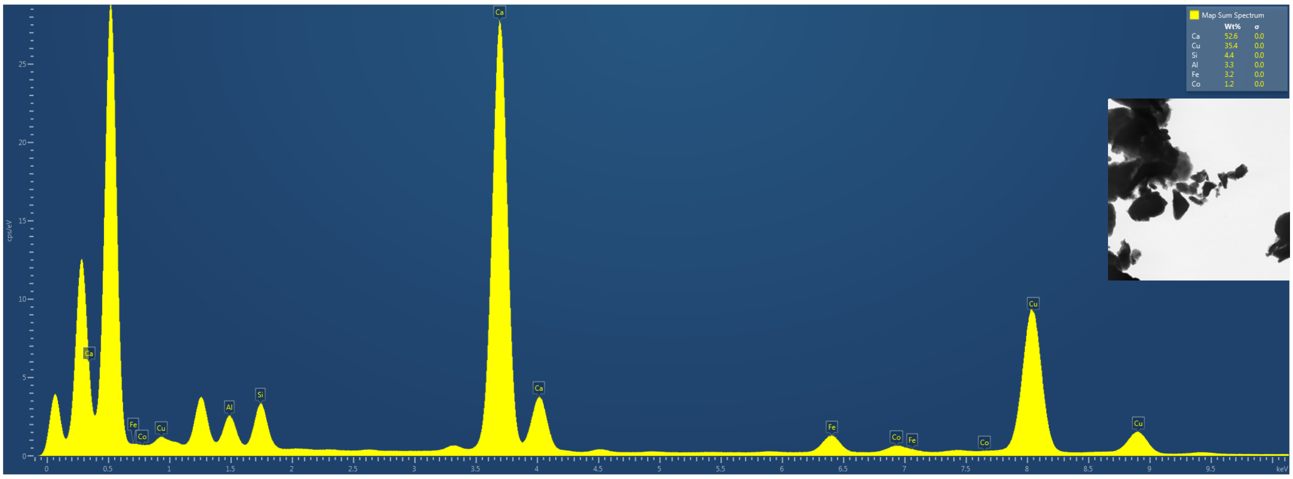


### (b) Belt entry

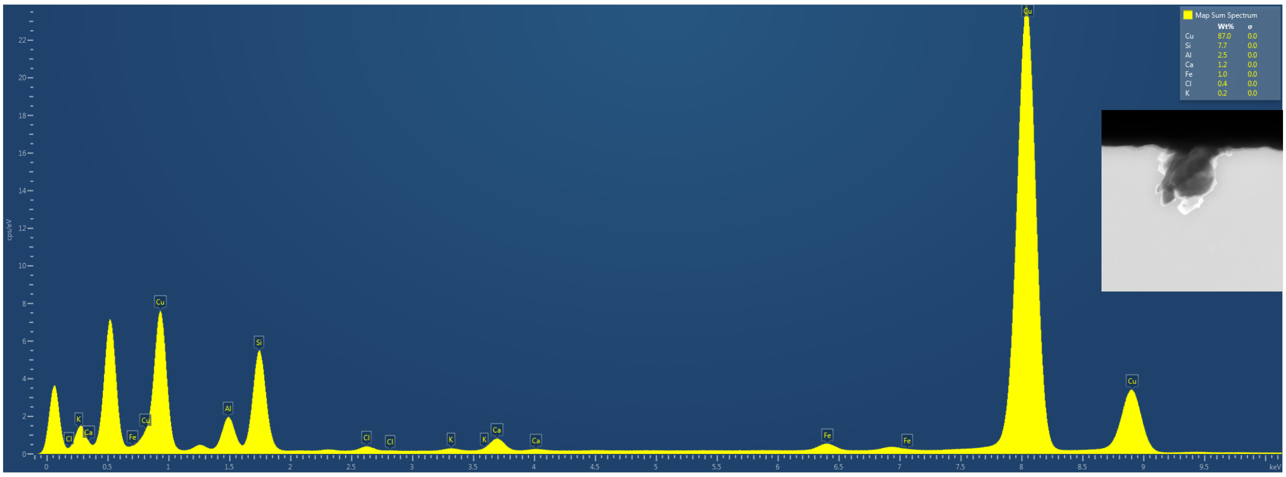


### (c) Belt conveyor drift area

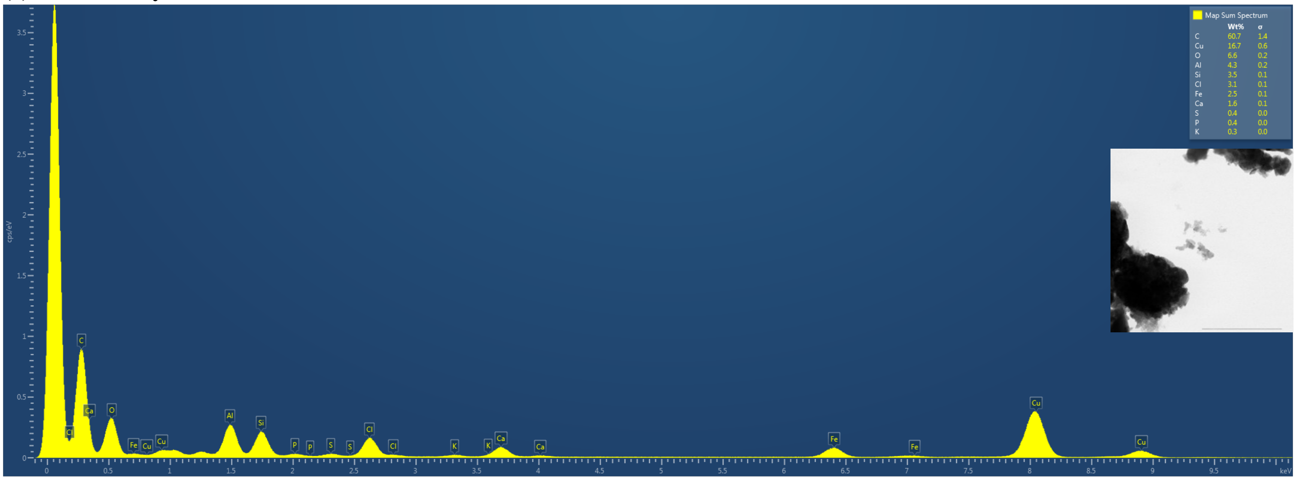

**(d) Personal**

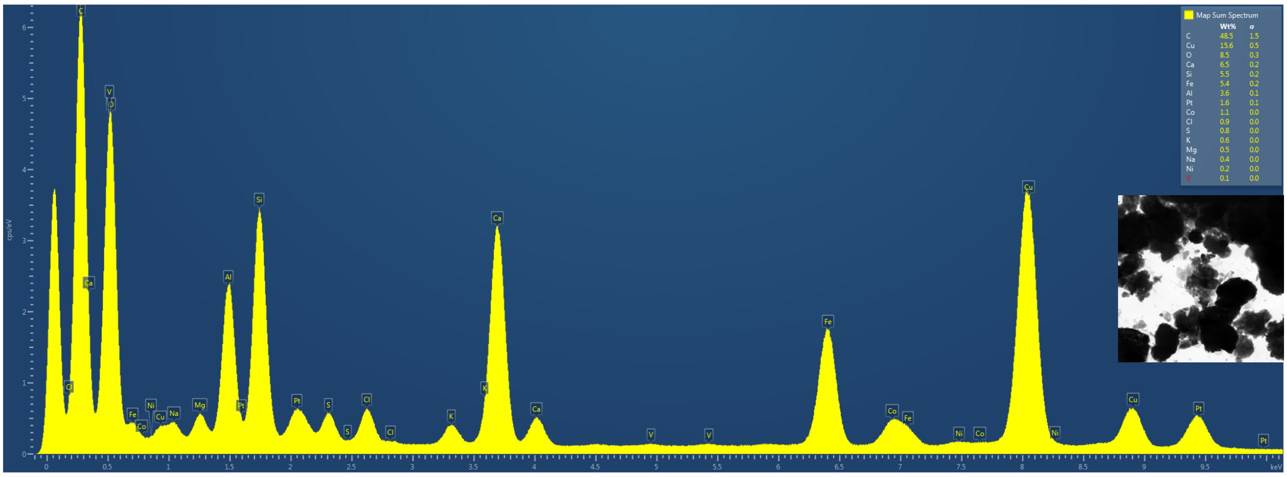


SI 1. Elemental compositions of dust particles sampled from the underground mine in multiple locations by STEM with EDS. (a) Office building; (b) Belt entry; (c) Belt conveyor drift area; (d) Personal at belt conveyor drift area.
